# Strengthening of Reinforced Concrete Non-Circular Columns with FRP

**DOI:** 10.3390/ma16216973

**Published:** 2023-10-30

**Authors:** Yavuz Yardim, Salih Yilmaz, Marco Corradi, Waleed A. Thanoon

**Affiliations:** 1 Department of Civil and Environmental Engineering, The University of Edinburgh, Edinburgh EH9 3FB, UK; yyardim@ed.ac.uk; 2 Earthquake Engineering Research Center, Izmir Katip Celebi University, Cigli, Izmir 35570, Turkey; thesalihyilmaz@gmail.com; 3 Department of Engineering and Technology, University of Huddersfield, Huddersfield HD1 3DH, UK; 4 Department of Civil Engineering, Zarqa University, Zarqa 13110, Jordan; thanoonwaleed@yahoo.com

**Keywords:** confinement, composite materials, reinforced concrete, FRP anchors

## Abstract

Fiber reinforced polymer (FRP) strengthening in circular columns is known to be more effective than in rectangular and square columns because of the uniform distribution of confining pressure. This study explores the effectiveness of using carbon-FRP anchors to improve the confinement of square reinforced concrete (RC) columns strengthened with FRP. Sharp corners in non-circular columns cause stress concentration on the corners, reducing the effectiveness of strengthening. To address this, the study examines the impact of three different anchor configurations on two sizes of FRP-strengthened square columns. The results show that the proposed anchors distribute stresses to a greater extent, resulting in a more uniform distribution of stresses and better confinement. For the best performance, it is proposed that the anchor fans surround the corners of the cross section. Experimental findings and finite element analysis results using the Concrete Damage Plasticity model in the ABAQUS material library match.

## 1. Introduction

It is widely recognized that concrete buildings constructed in the past do not perform satisfactorily under extreme loading conditions [1]. In addition, the use of low-quality materials [2] and poor workmanship have significantly impacted the structural performance of concrete buildings. Consequently, different techniques have been proposed to improve the structural capacity and strengthen existing concrete structures [3]. In this context, reinforced concrete columns are the primary structural elements that assure stability, as their collapse can cause the entire concrete structure to fail [4]. Various techniques have been used to enhance the overall capacity of reinforced concrete columns, including ferrocement, steel jacketing, and FRP strengthening [5,6,7,8,9] using composite sheets and plates. FRP sheet wrapping is the most recent and preferred strengthening system, offering a combination of desired mechanical characteristics achieved at a reasonable cost and ease of application [3]. Though FRP sheet wrapping could already achieve good results, FRP column strengthening may have further potential for improvement by introducing better confinement schemas. For improving column capacity, it is important to consider the concrete strength that controls the curvature demand and the axial capacity of the column. Providing lateral confinement can significantly increase the compressive strength and ductility of concrete. This allows the concrete core to carry additional compressive stress and increase its compressive capacity.

Concrete column confinement with FRP sheets has provided a considerable increase in axial [10,11,12,13,14,15,16,17,18], flexural strength [19], and deformation capacity. Previous research has shown that the increase in axial load capacity of columns varied between 6% and 177% [20]. The tensile strain capacity of FRP fibers is not fully occupied, thus the circumferential ultimate strain of the column to the ultimate strain capacity of FRP ranges from 0.55 to 0.62 [21]. This is further compounded by the aspect ratio factor in rectangular or square columns, where stress concentrates in the corners [14]. As a result, the effective strain coefficient is substantially reduced, leading to lower strength enhancement despite the use of the same confinement ratio [22].

Various parameters of FRP strengthening systems were tested in previous research, such as the amount and orientation of the composite fibers [23], the concrete compressive strength, and the corner radius (for non-circular columns) on the effectiveness of confinement [19,24]. Also, the type and mechanical properties of the FRPs have been investigated; carbon, glass, aramid, and basalt fibers are the main types of composites used to confine concrete columns [25].

Typically, circular sections result in confining forces or pressure producing a uniform stress distribution along the concrete section perimeter, while non-circular sections have different stress profiles due to the inability of the confining material to restrict displacement outside the central region of the concrete material. To reduce the problem of non-uniform stress distribution in non-circular columns, researchers have focused on modifying the concrete cross section. This can be achieved by chamfering the corners to a desired radius [19] or by adding material to change the rectangular shape to a circular or elliptical one, using expansive cement grout. The most common method for strengthening these columns is to chamfer the corners with radii of 20 mm to 30 mm before applying FRP sheets. In order to achieve a greater capacity increase, larger chamfer radiuses (from 35 to 45 mm) or high confinement ratios (up to 1.5 mm layer thickness) have been used [13,26,27].

Though increasing the corner radius provides better FRP confinement, it is limited by the presence of existing reinforcement, as early code provisions call for smaller concrete covers. In addition, FRP usage is restricted for lower-grade concrete (typically C12/15 and C16/20 strength classes) according to design codes, respectively [28,29]. An alternative approach is needed to confine non-circular columns effectively and achieve a more even stress distribution while keeping the corner radii and confinement ratio within acceptable limits.

This study proposes carbon fiber reinforced polymers (CFRP) anchors in low confinement areas to enhance the efficiency of FRP strengthening in non-circular columns. The system evenly distributes stresses across the column, improving confinement during concrete core dilation and increasing axial capacity. Bonding between anchors and concrete using epoxy offers significant tensile capacity and redistributes forces within the section. This study investigates the effects of three anchor configurations on FRP-strengthened square-section columns of varying sizes through experiments and finite element (FE) modeling with ABAQUS, version 2016.

## 2. Experimental Program

### 2.1. Specimen Details

The experimental program aims to investigate the effects of varying parameters on the behavior of confined concrete columns. The parameters include the specimen size, anchor layout, and number of anchors (Table 1). One CFRP unidirectional sheet is applied to the surface of all strengthened specimens. To establish a solid reference, the selection of specimen sizes is based on findings from the existing literature. A comprehensive database was used to set up the research objectives and the variables to consider in the experimental work [13,26,30]. The dimensions of the concrete specimens were 150 × 150 × 300 mm and 200 × 200 × 300 mm, where 300 mm is the height of the square specimens. The specimens’ size of 200 × 200 × 300 mm was tested to evaluate the potential influence of the sectional area to height ratio [30]. A total of 20 specimens were tested within the scope of this study. According to the European norms [31] and the ISO 1920-4 standard [32], a nominal length-to-diameter ratio equal to 2 is recommended. The height of the 15 cm square columns was 30 cm. To study the effect on less slender columns and to better compare test results by reducing the test variables, the height of 20 cm square columns was also 30 cm. When a nominal length-to-diameter ratio smaller than 2 is used, the compression test overestimates the concrete compressive strength by about 10–12%. However, since comparison is made within the same batch of samples (15 cm or 20 length concrete samples) in reinforced and unreinforced configurations, the length-to-diameter ratio does not significantly affect the results in terms of the increase in compressive strength due to reinforcement.

An increase in the corner radius of the concrete specimens is known to enhance the efficiency of confinement. Literature data suggest that the minimum corner radius for full-scale concrete columns should be 13 mm [28]. In this study, a conservative approach is taken, and a corner radius of 15 mm was used to represent a worst-case scenario.

**Figure 1:**
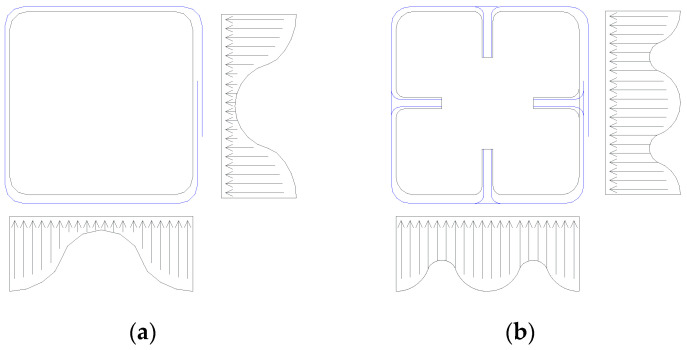
Pressure distribution in the confined section, without (**a**) and with (**b**) anchorage.

The anchors consist of a dowel, which transfers stress to the concrete core, and a fan that accumulates fiber stresses within the embedment depth and transfers them to the concrete core. The dowel part of the FRP anchor effectively simulates the confining behavior exhibited by steel hoops in reinforced concrete. Three types of FRP anchors were used in this study: simple anchorage (SA), corner anchorage (CA), and fan anchorage (FA) [33,34]. SA and CA aim at providing better confinement to the concrete core, whereas FA generates minimum disturbance to the column’s concrete core, which is the main part to carry the axial load. Hypothetical stress distribution aimed at being achieved through FRP anchors is visually depicted in Figure 1. 

**Figure 2:**
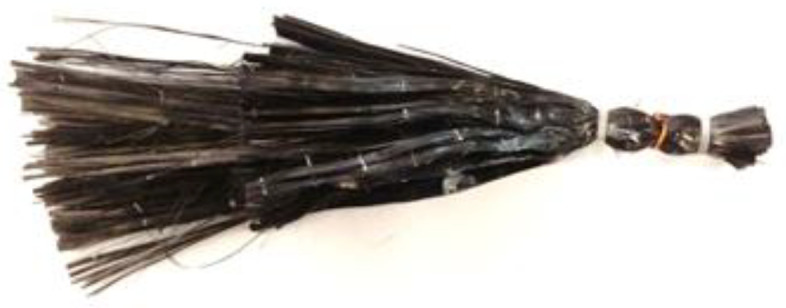
Detail of the CFRP anchors.

To prepare the anchors, FRP sheets of different lengths and 10 mm width were rolled, and the fibers were separated for better distribution along the surface. For dry anchor dowels, thin copper wires were wound around the fibers and secured with plastic clips to create a tube-like structure that can be inserted into the drilled holes (Figure 2).

**Figure 3:**
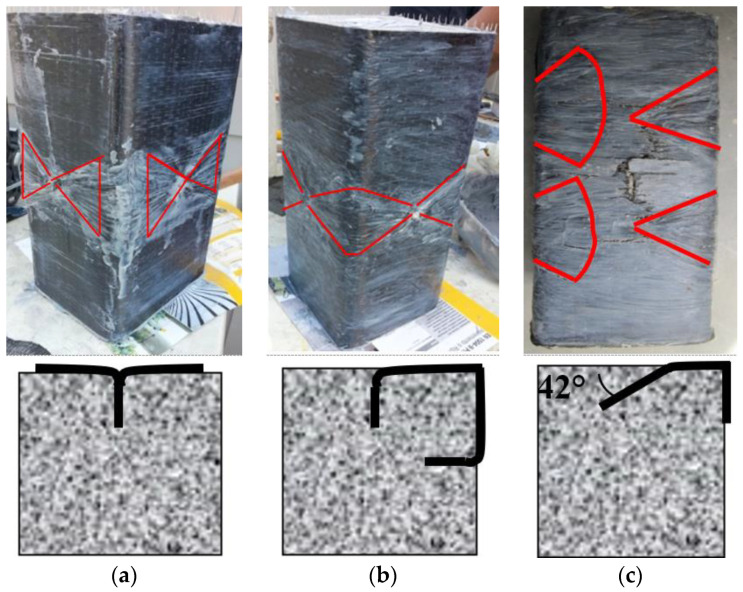
Anchorage layouts: (**a**) Simple; (**b**) Corner; (**c**) Fan.

The general properties of the anchors are given in Table 2. Simple anchors were just covering the corresponding surface; however, fan and corner anchors surrounded the corners. Therefore, fan lengths and corner anchors are selected accordingly. The typical hole angle for corners and simple anchors are selected as perpendicular to the surface, where openings are grained with a router bit to avoid a sharp corner. The finished applications of the three anchor types are shown in Figure 3. On the other hand, the drilling angle for fan anchors is 42° where no graining was applied (Figure 3c). 

### 2.2. Materials

To minimize the scattering of test results due to the variation in concrete strength, a cubic compressive strength of 25 MPa was selected, and ready-made concrete was used to cast the specimens, all of which were made using the same batch and mix design (Table 3).

**Figure 4:**
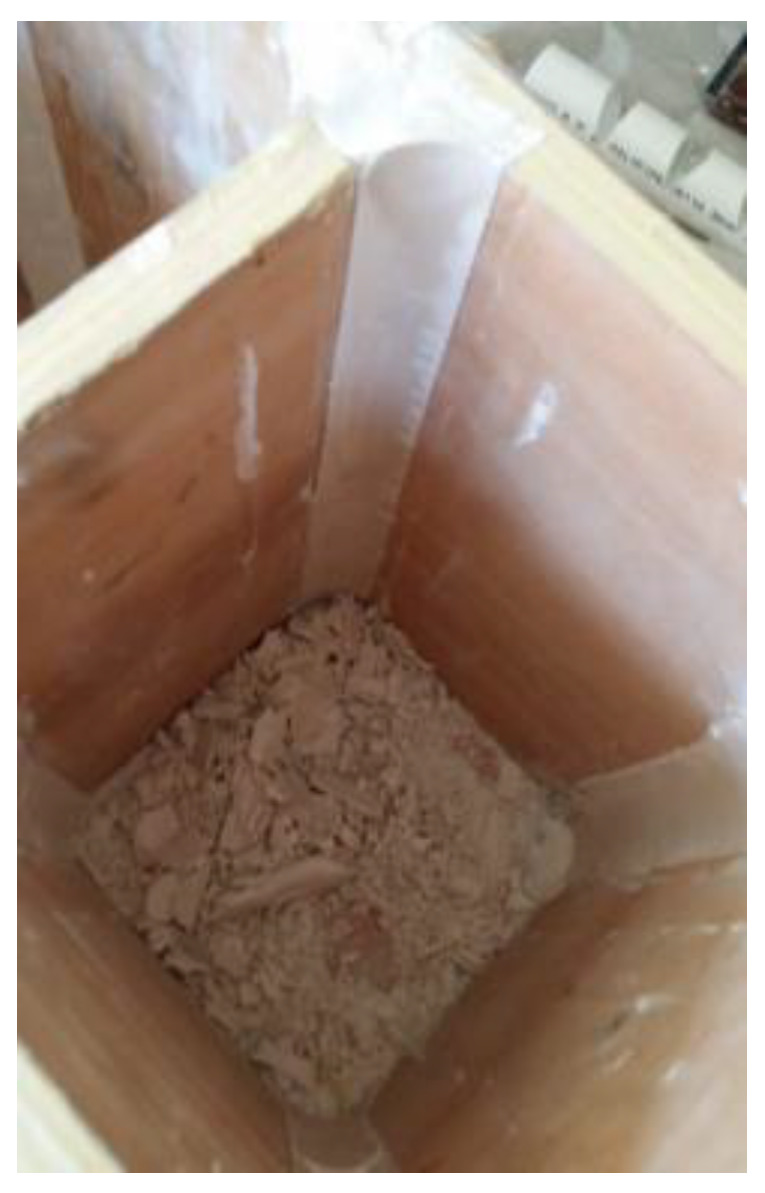
Plywood mold with rounded corners.

To achieve the desired rounded corners, modified plywood molds were used (Figure 4). Gypsum was used to fill the corners and provide a smooth surface. The specimens were cured for one week and then placed in a water tank for the remainder of the time. Then the specimens were confined with woven unidirectional SikaWrap-230 C fabric, which had an equivalent thickness of 0.129 mm and was impregnated with epoxy resin (Sikadur 330), produced by Sika Company, (Baar, Switzerland)

The adhesive used was a thixotropic, solvent-free, two-component impregnation resin based on epoxy with a tensile Young’s modulus of 4500 MPa (from the producer data sheet). Three concrete cubes (15 × 15 × 15 cm) and cylinders (15 cm in diameter, 30 cm in height) were tested on the same day as the FRP-wrapped specimens to compare the unconfined and confined compressive strengths. After 28 days, the mean compressive strength of the confined concrete cubes was 29.1 MPa. Table 4 lists the mechanical properties of the CFRP sheet provided by the manufacturer.

### 2.3. Specimen Preparation and Test Setup

**Figure 5:**
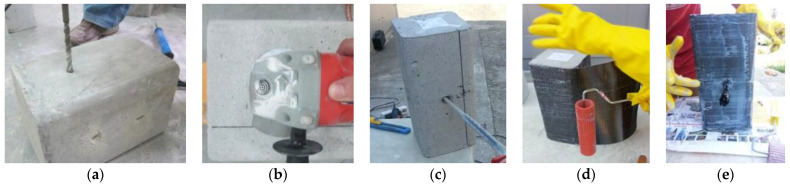
FRP and anchorage application method: (**a**) Drilling, (**b**) Grinding, (**c**) Filling holes with epoxy, (**d**) Apply FRP layer over epoxy, (**e**) Anchorage application.

Initially, the surface of concrete samples was treated with a concrete grinding machine to improve the adhesion of epoxy to the concrete. After grinding, the aggregate surface was visible, revealing the underlying voids, which were filled with epoxy. Holes were drilled on every specimen with a particular layout to place the anchors. The holes were chamfered with a needle scaler to avoid 90° bending of FRP anchors. The anchorages were placed in the center of the concrete column cross-section in order to evenly distribute the stresses from the FRP throughout the column. This helps to prevent the column from cracking or failing under load. The reinforcement procedure is shown in Figure 5.

**Figure 6:**
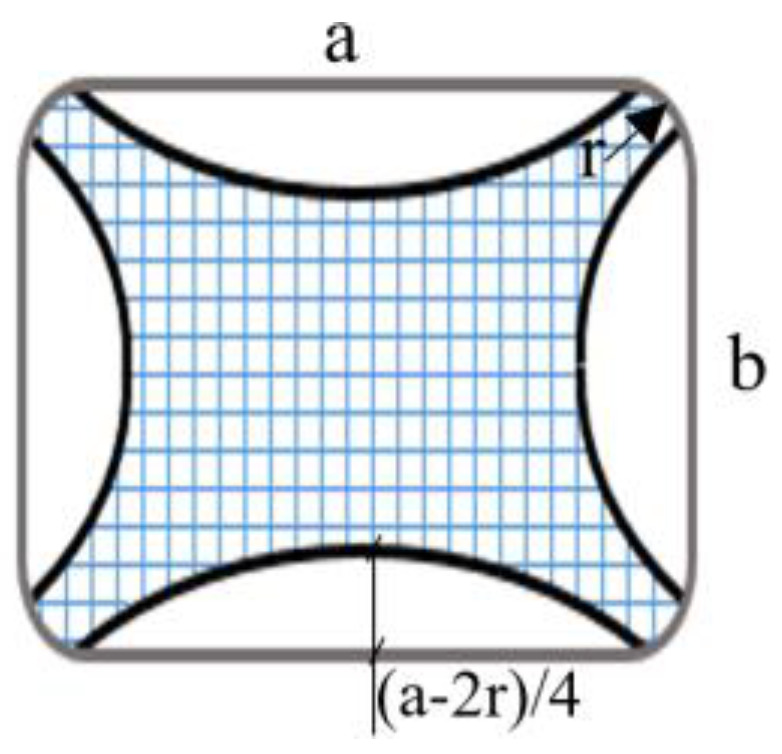
Effectively confined area (core section) of the square cross section.

The shaded area shown in Figure 6 is confined, with a maximum confinement depth of 3 cm and 4.25 cm [(a − 2r)/4] for 15 × 15 cm and 20 × 20 cm specimens, respectively. After drilling the holes, dust was removed from the hole before filling the epoxy with compressed air. A resin gun was used for epoxy application. The FRP anchors were embedded, and the anchors on the neighboring surfaces completely overlapped each other [34].

The FRP sheet was placed over epoxy using the hand layup method. The dowel was inserted through the FRP sheet and epoxy hole with minimal disruption to the fibers (Figure 5). The anchor fan was split in half with a dowel in the hole, then spread at a 30 ° angle from the anchor center towards the column’s sides.

**Figure 7:**
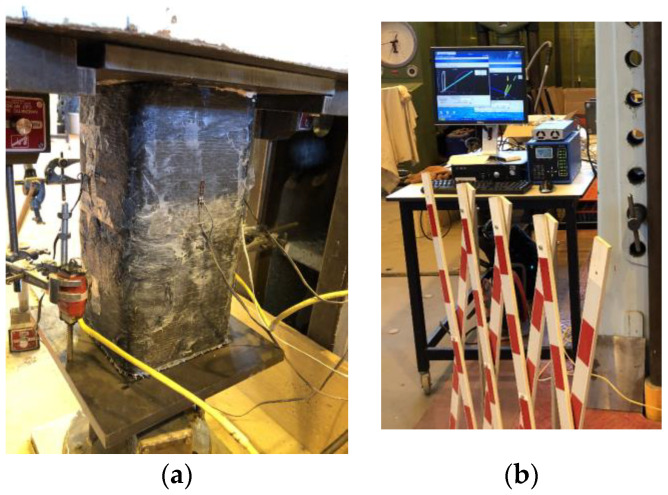
A reinforced concrete sample under compressive testing (**a**) and an acquisition system (**b**).

All specimens were tested under uniaxial compression until failure (Figure 7). The loading rate was 15 ± 3 MPa/min. Two linear variable displacement transducers were used to measure axial strains, placed 180° apart between the upper and lower loading plates.

## 3. Test Results

**Figure 8:**
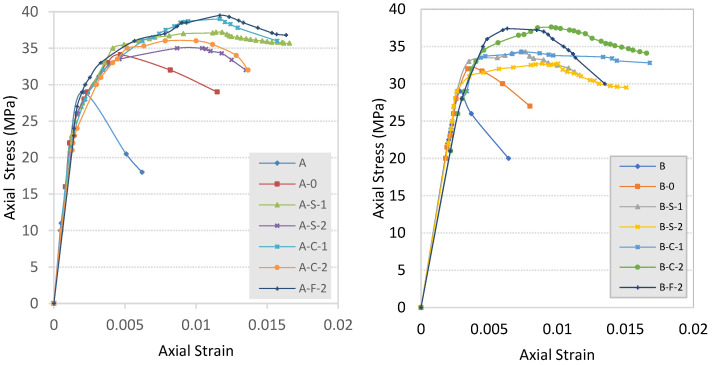
Stress–strain of anchored, un-anchored, and plain concrete specimens.

The tests showed that all three-anchor schemas increased the axial capacity and improved the ductility of the confined concrete column samples. The results followed the same path for both small- and large-size specimens. Results in terms of stress and strain values are summarized in Table 5 and shown in Figure 8.

### 3.1. Compressive Load Capacity

The experimental results demonstrated that CFRP-confined columns exhibited superior capacity performance compared to unconfined ones. Specifically, the axial capacity increased by 23.15% and 14.29% for specimens A-1 and B-1, with corresponding axial strain increases of 2.7 and 2 times, respectively. Further enhancement of compressive capacity was achieved by using additional anchors with CFRP wrap, with improvements ranging from 2.94% to 17.5%. Among all the specimens tested, those where the anchorage extended beyond the corner displayed the highest contribution to compressive capacity. Notably, fan anchorages demonstrated better confinement for square columns and exhibited a larger axial load-carrying capacity than corner anchorages. For the B and A test series, the capacity of the fan-anchored confined column showed a 17.5% and a 16.17% increase, respectively, in comparison to their un-anchored counterparts. Detailed results are presented in Table 4 and Table 5. A-C-2 had a lower capacity than A-C-1 due to the eight anchor holes weakening the limited core section of the specimen. The performance of fan anchors being better than corner anchors, which fully cover the corner from both sides, was unexpected. This probably depends on two reasons: (1) an inclined drilling angle providing no corner effect at hole opening; and (2) no disturbance of the concrete core by the inclined hole schema. 

The reduction in compression capacity in double-anchored specimens revealed that disproportionate disturbance resulted in lower levels of capacity improvement. On the other hand, A-F-2 and B-F-2 samples had eight anchor holes as well. However, the inclined orientation of the anchors did not disturb the core section of the specimen (Figure 3), resulting in better compression capacity.

### 3.2. Failure Modes

**Figure 9:**
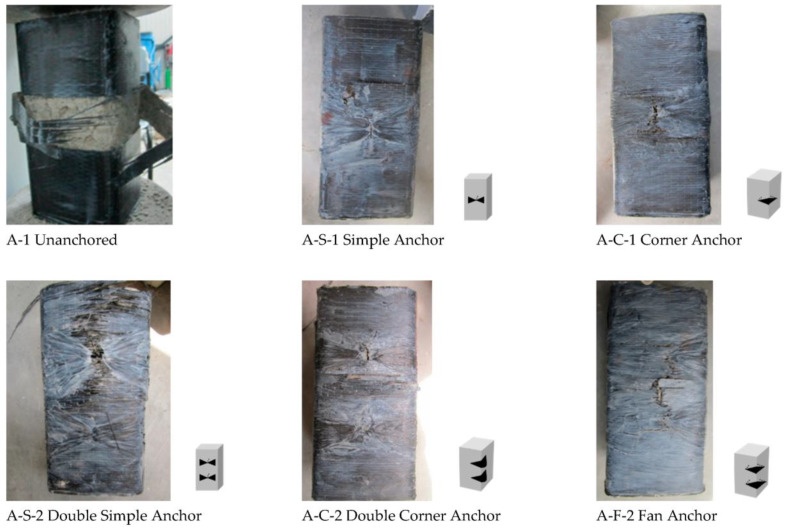
Experimental results: failure mode for specimen series 15 × 15 × 30 cm.

**Figure 10:**
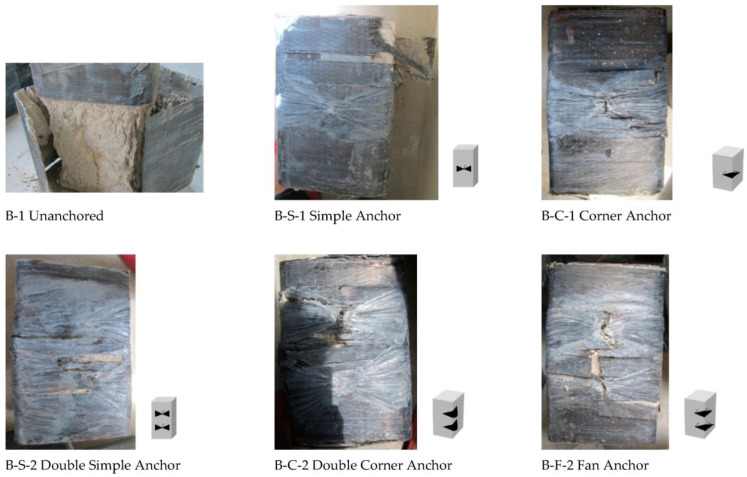
Experimental results: failure mode for Specimen Series 20 × 20 × 30 cm.

**Figure 11:**
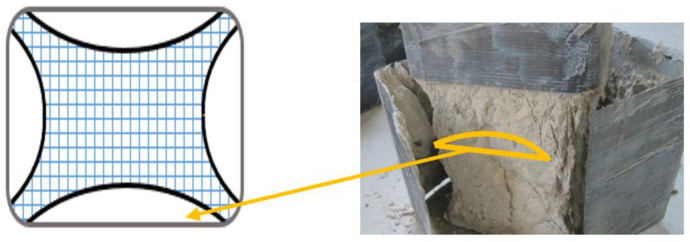
Failure of concrete in the least confined area.

The CFRP jacket fractured mostly near the corner due to stress concentration in un-anchored specimens (A-1/B-1). Confined specimens had CFRP tension failure at mid-height of corners (Figure 9 and Figure 10), causing bulging of the concrete core at the least confined section. The core retained the shape of the confined area prescribed by the parabolic lines (Figure 11). This explains the non-uniform confining pressure distribution. This strain, stress, and failure mode scenario can explain the non-uniform confining pressure distribution.

The smaller samples (A) failed in the middle, above or below the anchor dowel. The larger samples (B) had different failure modes depending on the type of anchorage. Samples strengthened with single and double simple anchorage (B-S-1 and B-S-2) failed at the corners, while samples strengthened with corner (B-C-1, B-C-2) or fan (B-F) anchorage failed in the middle. The different failure modes were due to the different fan lengths of the two sets of specimens. The simple anchorage contributed more to the failure mode for the A series, as it acted partially as a corner anchorage for the smaller specimens. In summary, the uniformity of the confining pressure increased with the anchorages, and it was more significant for the specimens strengthened with the corner anchorage for both test series.

All anchored specimens in this experimental study exhibited little or no strain-softening behavior (f_cu_/f_cc_ > 90%). In contrast, un-anchored specimens showed considerably lower ultimate loads when compared to their peak strength (f_cu_/f_cc_ = 68.4% for a small specimen). Table 5 summarizes the results in terms of strain-softening behavior from the experimental work. The performance of the anchored specimens provides evidence that the existence of an anchor enhances the uniform distribution of confining pressure.

## 4. Numerical Analysis

### 4.1. Modeling

This study used finite element analysis with the ABAQUS package to model the nonlinear behavior of un-anchored and single-corner anchor (A-C-1) 15 × 15 × 30 cm column specimens. To reduce model size, only one-quarter of each column section was modeled along its longitudinal axis. Symmetrical boundary conditions were assigned, and axial loads were applied using a displacement control method. Experimental results in this study show that the anchors were firmly attached to the concrete core and did not separate from it under any failure behavior. Therefore, it was concluded that they were rigidly attached to the concrete core. 

Previous research has shown that actively confined and FRP-confined concrete have different plastic behaviors. FRP-confined non-circular sections have complex and non-uniform stress variations, but experimental and FE results agree on the general behavior and stress–strain curves of FRP-confined columns [35]. This study found that FRP-confined square columns with anchors achieve a more uniformly distributed stress than those without. The ABAQUS material library’s Concrete Damage Plasticity model, which can represent both tension cracking and compression crushing, was used to model confined concrete. This study assumed a Poisson’s ratio of 0.2 for concrete.

The Young’s modulus was calculated using the ACI formula [24,28] as shown in Equation (1), and compressive strength (${f’}_{c}$) was found experimentally in MPa.

(1) $$E_{0}=4734\sqrt{{f’}_{c}}$$

The uniaxial tensile strength *f_t_* of the concrete was taken as in Equation (2)

(2) $$f_{t}=0.33\sqrt{{f’}_{c}}$$

The concrete under uniaxial compression is described by the stress–strain relationship proposed by Saenz [36] and given in Equation (3):

(3) $$\sigma_{c}=\frac{E_{0}\varepsilon_{c}}{1+\left(\frac{E_{0}\varepsilon_{p}}{\sigma_{p}}-2\right)\left(\frac{\varepsilon_{c}}{\varepsilon_{p}}\right)+{\left(\frac{\varepsilon_{c}}{\varepsilon_{p}}\right)}^{2}}$$

where *σ_c_* and *ε_c_* are the compressive stress and strain, respectively, and *σ_p_* and *ε_p_* are the experimentally determined maximum stress and its corresponding strain, which are obtained from standard cylinder tests. 

**Figure 12:**
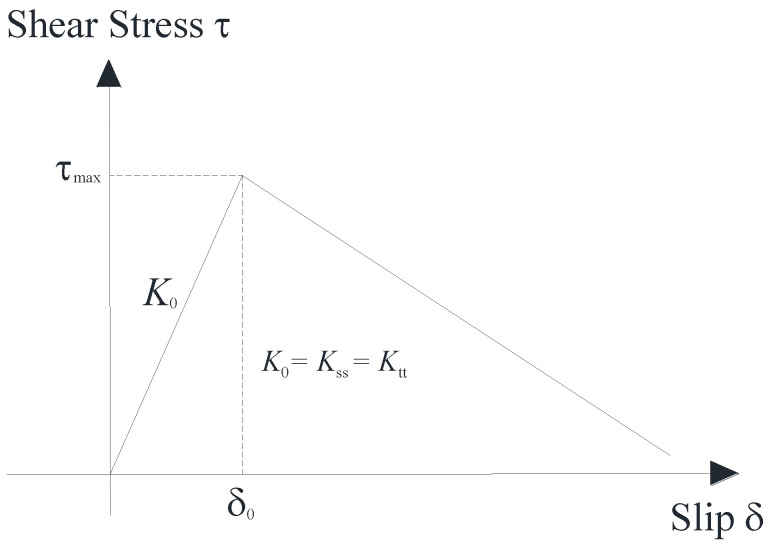
Bilinear traction separation constitutive curve.

The interface between concrete and CFRP was modeled using the cohesive zone model representation. A traction-separation model was used to represent the interface, assuming an initial linear elastic behavior with a stiffness K evolution of damage (Equation (4)). The model was interpreted in ABAQUS using a bilinear traction separation constitutive curve (Figure 12) in terms of effective traction (*τ*) and effective separation (δ). Elasticity is defined by nominal strain and tractions, using an elastic constitutive matrix (Equation (5)) to show the behavior of each traction component.

(4) $$\tau_{max}=1.46{G_{epoxy}}^{0.165}f_{ct}^{1.033}$$

where *G_epoxy_* is the shear modulus of adhesive in GPa and *f_ct_* is the tensile strength of concrete in MPa.

(5) $$\left\{t_{n}\;t_{s}\;t_{t}\;\right\}=\left[K_{nn}\;0\;0\;0\;K_{ss}\;0\;0\;0\;K_{tt}\;\right]\left\{\varepsilon_{n}\;\varepsilon_{s}\;\varepsilon_{t}\;\right\}$$

where

(6) $$K_{nn}=\frac{1}{\frac{t_{c}}{E_{c}}+\frac{t_{epoxy}}{E_{epoxy}}}$$

(7) $$K_{ss}=K_{tt}=\frac{1}{\frac{t_{c}}{G_{c}}+\frac{t_{epoxy}}{G_{epoxy}}}$$

*G_c_* is the shear modulus of concrete in MPa, *G_epoxy_* is the shear modulus of epoxy in MPa, *t_epoxy_* is the epoxy thickness (1 mm), *t_c_* is the concrete thickness (5 mm), *E_c_* is the Young’s modulus of concrete in MPa, and *E_epoxy_* is the Young’s modulus of the adhesive in MPa.

### 4.2. Analysis Results

**Figure 13:**
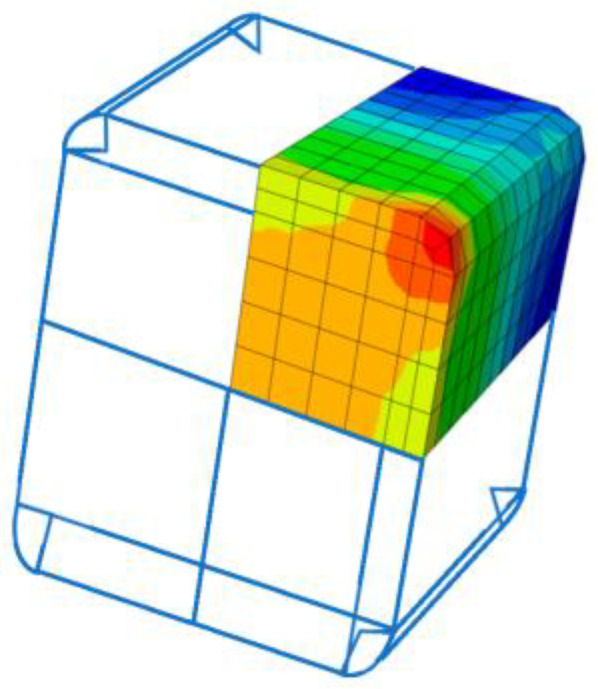
Detail of the well-known problem of stress concentration at corners. To reduce this, corners were rounded in this experimental work.

Figure 13 shows the stress concentration near the corners of the square concrete samples. This is a well-known problem, highly reducing the effectiveness of FRP wrapping on concrete columns. FRPs exhibit very low shear strength, and FRP fractures often develop from the corners. By rounding the corners, it is possible to reduce the negative effect of the corners. 

**Figure 14:**
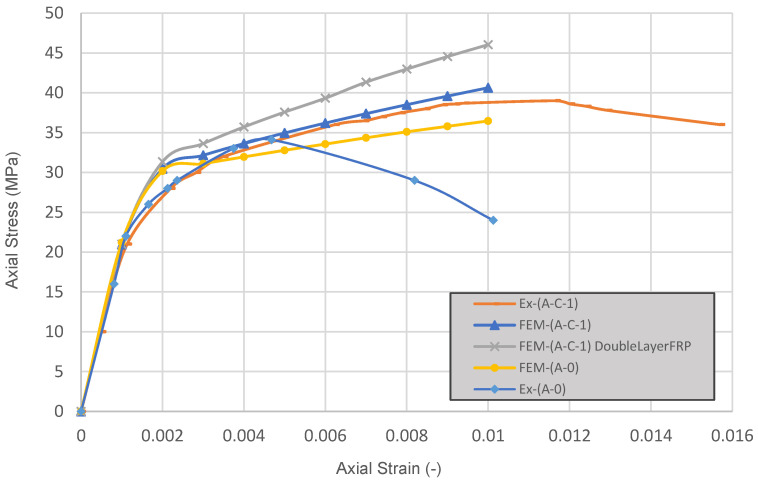
Compressive axial stress vs. axial strain of the A-C-1 and A-0 specimens: experimental vs. ABAQUS model.

Stress–strain curves obtained by FE analysis were plotted together with the experimental results in Figure 14 to validate the FE model. The figure shows the axial stress–strain relationship of non-anchored, single-anchored, and single-anchored with double-layer FRP-wrapped columns, along with the experimental results of single-anchored columns (A-C-1). Additionally, the axial stress–strain relationship of a column with double-layer FRP and single anchorage was also added to check the effects of thicker FRP layers on the curves.

Overall, the FE analysis approach used in this study is in good agreement with the experimental results of anchored and un-anchored specimens. The damaged plasticity model shows better agreement with FRP-confined columns where anchorages were used.

**Figure 15:**
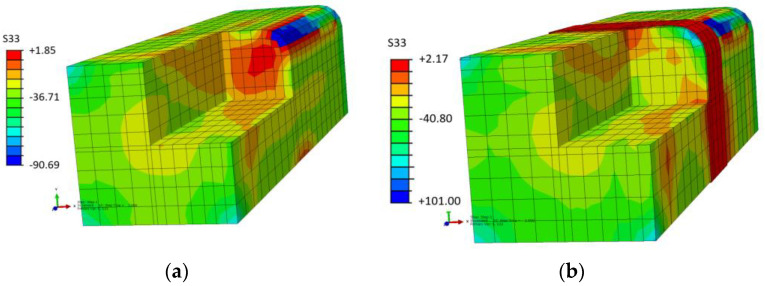
Compressive stress distribution S33: (**a**) Un-anchored; (**b**) Single-anchored specimen. Units: MPa.

The influence of confining pressure and anchorage on axial stress capacity and stress distribution was investigated, with hydrostatic pressure playing a significant role. It was observed that failure occurred on the plane above the anchorage, which was analyzed subsequently. The sectional distribution of confining pressure in un-anchored and single-anchored specimens is shown in Figure 15. The un-anchored specimen showed the highest level of confining pressure in the corner of the column, whereas the anchored specimen had pressure distributed all around the section’s perimeter. These findings have important implications for the design and construction of FRP-confined columns.

The S33 values in the legend box in Figure 15 are the axial compressive stresses on the concrete. A notable change in the axial stress over the section is shown. The anchorage provides a higher axial stress capacity and a more uniform stress distribution over the section. The anchored specimens’ performance shows that an anchor generates a uniform distribution of confining pressure.

This study aimed to reduce confining stress concentration in corners of FRP-confined prismatic column sections. To achieve this, FRP anchors were positioned on the faces with the least confinement and extended to the corners to distribute the stress more uniformly. This study evaluated three distinct anchorage layouts on two sizes of square column samples. The anchors improved the load-bearing capacity of the columns and prevented premature failures of the corners.

Compared to columns without anchorage, the axial compression capacity of confined column specimens was increased by up to 17.5% when FRP anchorage was used. It is noteworthy that a significant increase in compressive strength was observed in the areas where the anchorages surrounded the corner, such as with the fan and corner anchorage. This figure clearly shows that simple FRP anchor application does not improve axial capacity, nor does confinement like steel crossties in reinforced concrete. Rather, FRP anchors should surround the corners of the member.

Only a slight increase in axial strength was observed for double simple anchorage samples. This result may have been affected by the presence of eight anchor holes in a small cross-sectional area, which produced a weaker concrete core. To better understand the confining mechanism of multi-anchorages, a larger-scale specimen should be tested.

The use of anchors resulted in a reduction in strain-softening behavior. The column specimens anchored with different anchorage layouts showed almost no strain-softening behavior. The confinement kept the concrete core intact beyond the peak load until rupture. The majority of the anchored FRP-confined specimens failed in the mid-face, indicating that the failure mode was directly influenced by the stress distribution mechanism of the anchor. This mechanism redistributed stress and provided a uniform confining pressure around the section.

The finite element analysis approach used in this study is in good agreement with the experimental results of both anchored and un-anchored specimens. The analysis results indicate that the anchorage provided a higher axial stress capacity and a more uniform stress distribution over the section. These findings are in line with the experimental data. It can be highlighted that the results of this study have a number of implications for practice. First, this study shows that FRP anchors can be used to improve the confinement of FRP-strengthened square columns, where chamfering on corners is limited by rebar. Additionally, the required number of confining layers can also be reduced by introducing FRP anchors.

## 5. Conclusions

This study explores the effectiveness of using CFRP anchors to improve the confinement of square RC columns strengthened with CFRP sheets by wrapping. Sharp corners in non-circular columns cause stress concentration on the corners, reducing the effectiveness of strengthening. To address this, the study examines the impact of three different anchor configurations on two sizes of FRP-strengthened square columns. Three types of CFRP anchors were used in this study: simple, corner, and fan anchorage. Simple and corner types aim at providing better confinement to the concrete core, whereas fans generate minimum disturbance to the column’s concrete core, which is the main part to carry the axial load. An increase in axial load capacity ranging between 14% and 20% was recorded for concrete samples reinforced without anchors (only CFRP wrapping). The application of a fan anchorage improved further the axial load capacity of the concrete samples, up to 39% compared to the control unreinforced specimens. 

Another interesting effect of the application of CFRP reinforcement is the improved post-elastic residual capacity after cracking. While the axial load capacity of unreinforced concrete specimens reduced dramatically after the peak load, reinforced ones were able to sustain a residual capacity up to 90% of the maximum compressive load. This was particularly noted for concrete specimens where anchors were in place. 

The results also show that the proposed anchors distribute stresses to a greater extent, resulting in a more uniform distribution of stresses and better confinement. For the best performance, it is proposed that the anchor fans surround the corners of the cross section. To achieve this, CFRP anchors were positioned on the faces with the least confinement and extended to the corners to distribute the stress more uniformly. On the other hand, it should be avoided to disturb core concrete. In order to achieve that, inclined drilling of anchor holes is a good practice. The authors suggest extending the study to rectangular columns, in which confinement by FRP wrapping is less effective with respect to square columns.

On the other hand, this study has a few limitations. Only one or two concrete samples were tested for each reinforcement and a control. This makes conclusions difficult. Concrete is variable, and a difference in the control and treatment can be attributed to this variability and not necessarily to the reinforcement. However, several similar reinforcement layouts have been tested, for a total of 20 compression tests, and all of them produced an improvement in the axial load capacity of the concrete columns. This is a clear indication of the effectiveness of the reinforcement methods. More tests will be necessary to assess the effect of the anchors. 

The finite element analysis conducted in ABAQUS solidified our understanding of the anchors’ impact in FRP-confined square columns, aligning well with experimental findings. The results from the numerical simulations played a crucial role in validating the enhanced load-bearing capacity and more uniform stress distribution achieved through the use of anchors. This validation was paramount in illustrating the reduction in stress concentrations around corners, an inherent issue in non-circular columns. The integration of these findings into our conclusions strengthens the case for implementing anchors in practical applications, ensuring a robust and well-supported set of design recommendations.

In addition, the investigation only involved FRP-strengthened square columns. It would be interesting to investigate the effectiveness of FRP anchors on rectangular columns. Second, this study only tested two sizes of columns as well. It would be interesting to consider the effect of FRP anchors on real-scale columns. Despite these limitations, this study provides valuable insights into the effectiveness of using FRP anchors to improve the confinement of FRP-strengthened square columns. The results of this study suggest that FRP anchors are promising for further improving the performance of FRP-strengthened columns. Future research should focus on investigating the effectiveness of FRP anchors on different shapes and sizes of columns, as well as the long-term performance of FRP anchors.

## Figures and Tables

**Table 1:** Test matrix.

Sample Dimensions (cm)	Series	No. of Tested Samples	FRP Anchor Type	No. of Anchors per Face
15 × 15 × 30	A-0	1	Unreinforced	
15 × 15 × 30	A-1	1	Reinforced without anchorage	
15 × 15 × 30	A-S-1	1	Simple	1
15 × 15 × 30	A-C-1	2	Corner	1
15 × 15 × 30	A-S-2	1	Simple	2
15 × 15 × 30	A-C-2	2	Corner	2
15 × 15 × 30	A-F-2	2	Fan	2
20 × 20 × 30	B-0	1	Unreinforced	
20 × 20 × 30	B-1	1	Reinforced without anchorage	
20 × 20 × 30	B-S-1	1	Simple	1
20 × 20 × 30	B-C-1	2	Corner	1
20 × 20 × 30	B-S-2	1	Simple	2
20 × 20 × 30	B-C-2	2	Corner	2
20 × 20 × 30	B-F-2	2	Fan	2

**Table 2:** Sizes and dimensions of the anchorages.

	Simple Anchorage		Corner Anchorage		Fan Anchorage	
Concrete Sample Dimensions (cm)	15 × 15 × 30	20 × 20 × 30	15 × 15 × 30	20 × 20 × 30	15 × 15 × 30	20 × 20 × 30
Fan Length (cm)	6.5	7.5	15	20	8	12
Hole Depth (cm)	4	5	4	5	7	7
Hole Diam. (cm)	1.2	1.2	1.2	1.2	1.2	1.2
Hole Angle to Concrete Face (°)	90	90	90	90	42	42

**Table 3:** Concrete mix design.

Water (kg/m^3^)	200
Portland cement (kg/m^3^)	290
Cement Type	CEM II/A-L 32.5R
Fine aggregate (kg/m^3^)	930
Coarse aggregate (kg/m^3^)	940

**Table 4:** Mechanical properties of CFRP sheet (from producer data sheet, SikaWrap-230 C).

Fiber type	Carbon
Orientation	unidirectional sheet
Fiber dry weight density (g/m^2^)	230
Fiber tensile strength (MPa)	4300 *
Fiber Young’s modulus (GPa)	238 *
Fiber elongation at break (%)	1.8

* These mechanical values were calculated using an “equivalent thickness” of the unidirectional carbon sheet (0.129 mm).

**Table 5:** Experimental results of compression testing.

Series	*ε_co_* (-)	*f_cc_* (MPa)	*ε_cu_* (-)	*f_cu_* (MPa)	(*f_cc_* − *f_co_*)/*f_co_* (%)	(*f_cc_* − *f_o_*)/*f_o_* (%)	*f_cu_*/*f_cc_* (%)
A-0	0.0022	28.5 (*f_o_*)	0.0025	18.1	_	_	63.2
A-1	0.006	34.1 (*f_co_*)	0.0115	24.1	_	23.15	70.6
A-S-1	0.012	37.2	0.0166	35.7	9.41	30.52	95.9
A-S-2	0.0087	35.0	0.0135	32.9	2.94	26.31	94.2
A-C-1 *	0.010	39.0	0.0167	36.1	14.7	36.85	92.3
A-C-2 *	0.010	36.0	0.0137	32.0	5.88	24.56	88.9
A-F-2 *	0.012	39.5	0.0163	36.8	16.17	38.50	93.2
B-0	0.00255	28.1 (*f_o_*)	0.0064	19.1	_	_	67.9
B-1	0.0048	31.9 (*f_co_*)	0.0910	24.0	_	14.3	75.0
B-S-1	0.0062	34.1	0.0117	31.2	6.56	21.8	91.5
B-S-2	0.0089	32.7	0.0150	29.5	2.19	16.8	90.2
B-C-1 *	0.0074	34.9	0.0165	32.8	9.06	24.6	94.0
B-C-2 *	0.0095	37.3	0.0167	34.1	16.6	36.3	91.4
B-F-2 *	0.0080	37.6	0.0135	30.0	17.5	36.6	79.8

*ε_co_* Strain at Peak Load, *f_cc_* Stress at Peak Load, *ε_cu_* Ultimate Strain, *f_o_* Stress of unconfined specimen, *f_cu_* Ultimate stress, *f_co_* Stress of un-anchored FRP confined specimen. * average values.

## Data Availability

The data presented in this study are available on request from the corresponding author. The data are not publicly available due to privacy.
